# 

**Published:** 1820-02

**Authors:** 


THE LONDON

### Medical and Physical Journal.

2 OF VOL. XLIII.] FEBRUARY, 1820. [NO. 252.

"For many fortunate discoveries in medicine, and for the detection of numerous "errors, the world is indebted to the rapid circulation of Monthly Journals; "and there never existed any work to which the Faculty in EUROPE and "AMERICA were under deeper obligations than to the Medical and Physical "Journal of London, now forming a long, but an invaluable, series." RUSH.

### *ADDRESS.*

*WE should have apologized at the commencement of this Volume, for the omission of the usual Retrospect of Medical Literature. This, if applied to the progress of Medicine generally, we have found it impossible to continue without departing from the original and established Plan of the Journal; and to comprise merely a review of English Medical Productions must be nearly useless, because we omit but little of importance respecting them in the regular series of the Work. We have therefore let the Journal continue its former course, and produced a Supplement to the present Volume,* comprising a general History of Medicine during the last semi-annual period. We have thus left it to the option of our Readers whether or not they choose to obtain it: but, as it forms so essential a part of the Plan on which the Journal is now conducted, we should regret that the whole of them should not become possessed of it, as it comprises many important matters respecting the progress of Medicine in foreign nations, and general summaries of novel theories, which could not be inserted in the course of the last Volume.*

*In order to favor this plan, of comprising an extensive view of the progress of Medicine generally, we shall make another little modification in the regular series of the Work. The* Foreign Department *shall in future consist solely of critical Analysis, by which the observations and doctrines of Works may be much condensed; and, at the same time, nothing advanced positively without our opinion respecting its merit and truth. But, as many observations require detail, and many hypotheses are worthy of full narration, we shall occasionally insert complete relations of this kind, but place them with our national communications. We shall also, to further the same views, give, regularly, extracts from the Proceedings of Foreign Medical Societies, Reports of Hospital Practice, &c.; and by this method we expect, all this may be done without the omission of any thing of great importance that occurs in our own country.*

* Which was published in the latter part of January.

### MONTHLY CATALOGUE OF MEDICAL BOOKS.

The Army Medical Officer's Manual upon active Service; or Precepts for his Guidance in the various Situations in which he may be placed; with Observations on the Preservation of the Health of Armies upon Foreign Service. By J. G. V. Millinge n, M.D. Surgeon to his Majesty's Forces, &c. 8vo. boards, 9s.

Observations on the Use and Abuse of Mercurial Medicines in various Diseases. By James Hamilton, jun. M.D. 8vo. boards, 7s. 6d.

Remarks on the Cow-Pox; designed for general Reading. By Jonas Malden, M.D. 8vo. 1s.

Burgess and Hill's improved Medical Catalogue, containing the best Authors, with a Table of Contents, methodically arranged. 1s. 6d.

The Anatomist's Vade Mecum; containing the Anatomy, Physiology, Morbid Appearances, &c. of the Human Body; the Art of making Anatomical Preparations, &c. By Robert Hooper, M.D. 12mo. 8s.

